# Stress-Induced Increase in Cortisol Negatively Affects the Consolidation of Contextual Elements of Episodic Memories

**DOI:** 10.3390/brainsci10060358

**Published:** 2020-06-09

**Authors:** Matthew Sabia, Almut Hupbach

**Affiliations:** Department of Psychology, Lehigh University, Bethlehem, PA 18015, USA; sabiam3@gmail.com

**Keywords:** human memory consolidation, post-encoding stress, item-context binding, context memory

## Abstract

Stress can modulate episodic memory in various ways. The present study asks how post-encoding stress affects visual context memory. Participants encoded object images centrally positioned on background scenes. After encoding, they were either exposed to cold pressure stress (CPS) or a warm water control procedure. Forty-right hours later, participants were cued with object images, and for each image, they were asked to select the background scene with which it was paired during study among three highly similar options. Only male but not female participants reacted with a significant increase in salivary cortisol to CPS, and the stress and control group did not differ in recognition performance. Comparing recognition performance between stress responders and non-responders, however, revealed a significant impairment in context memory in responders. Additionally, proportional increase in cortisol was negatively correlated with the number of correctly recognized scenes in responders. Due to the small number of responders, these findings need to be interpreted with caution but provide preliminary evidence that stress-induced cortisol increase negatively affects the consolidation of contextual elements of episodic memories.

## 1. Introduction

Stress is a powerful modulator of learning and memory. Stress activates (a) the sympathetic nervous system, leading to a rapid release of catecholamines from the adrenal medulla, and (b) the hypothalamus-pituitary-adrenocortical axis, leading to a slower release of glucocorticoids such as cortisol from the adrenal cortex. Cortisol can pass through the blood–brain barrier and binds to mineralocorticoid receptors (MRs) and glucocorticoid receptors (GRs) that are expressed in brain areas crucially involved in learning and memory, particularly the hippocampus [1,2]. MRs have a high affinity to cortisol and are occupied under basal conditions. Stress-related increases in cortisol lead to the occupation of GRs, which directly affect hippocampal processes; for a review, see [3]. 

Stress can impair or enhance episodic memory, and the specific direction of influence depends on the interaction of many factors, including the intensity of the stress response, concurrent catecholamine activation, the hormonal state of the stressed individual, the emotionality and ecological validity of the memory content, and importantly, the timing of the stressor in relation to the memory stage. Most studies in humans have applied stress at encoding or before retrieval; for reviews, see [4,5]. Stress experienced before or during encoding selectively facilitates memory for stress-related or emotional experiences but impairs memory for stress-irrelevant or neutral information [6,7,8]. Stress negatively impacts retrieval of episodic memories; for reviews, see [5,9,10]. These effects support the view that stress puts memory into a formation mode, prioritizing the encoding and storage of ongoing stressful and emotional experiences over competing cognitive processes such as the recall of past events [4].

When stress occurs after encoding but well before retrieval as in the current study, effects reflect stress-related alterations of consolidation or forgetting rather than encoding and retrieval processes. In one of the first study on this issue, participants viewed a series of negative images before submerging their arm either in ice-cold water to induce cold pressor stress (CPS) or in warm water as a control procedure. One week later, participants were asked to recall the images in as much detail as possible. The stress group recalled significantly more arousing images than the control group, whereas the groups did not differ in the recall of non-arousing images [11]. Subsequent studies provided mixed findings with regard to the modulating effects of arousal and valence (for reviews, see [12,13]), but a recent meta-analysis showed that post-encoding stress generally improves episodic memory, irrespective of the emotionality of the learned material [5]. However, few studies have distinguished between different memory components and their underlying brain systems. As will be outlined in the following paragraphs, it appears that components of neutral memories that rely on the hippocampus are often negatively impacted by stress.

Studies employing recognition tests reveal that stress that is applied during the consolidation period primarily aids subsequent familiarity-based recognition [14,15], whereas recollection-based recognition is affected by stress-induced cortisol increase following an inverted U-shape function [16]. Recollection does not only involve the identification of a study item but also remembering the specific circumstances surrounding its presentation. This contextual signature is provided by the hippocampus, reflecting its unique ability to rapidly link different elements of an experience into a cohesive memory representation or event engram. Importantly, long-term retention of these engrams requires undisturbed hippocampal activity for a prolonged period of time after encoding; for a recent review, see [17]. Familiarity-based recognition, on the other hand, does not require the hippocampus and can be achieved by the perirhinal cortex alone [18,19]. The hippocampus is particularly sensitive to stress effects because of its high density of mineral and glucocorticoid receptors. While moderate stress can enhance memory by partially activating GRs, intense stress can cause GRs to become oversaturated, compromising hippocampal processing [3], consistent with the observed inverted U-shape function between cortisol and recollection.

Since the hippocampus plays a unique role in providing contextual signatures for memories, compromised hippocampal function can also contribute to false memories. Using the Deese–Roediger–McDermott (DRM) paradigm [20], Pardilla-Delgado et al. [21] showed that post-encoding stress decreased the recognition of target words and increased false recognition of lure words that were semantically related to the target words. Targets can be distinguished from semantically related lures by experimental context signatures, paralleling the distinction between recollection and mere familiarity-based recognition. Without access to these signatures, memory performance increasingly relies on gist information that is similar for targets and lures [22]. For emotional DRM lists, however, post-encoding stress can enhance target recall [23]. This difference is likely due to the selective involvement of the amygdala in emotional processing. The negative impact of elevated levels of cortisol on hippocampal-based processing can be offset by noradrenergic-induced potentiated activity in the amygdala that ensures the retention of emotionally salient information [24,25,26]. Not all forms of source memory are negatively affected by post-encoding stress. One study found that post-encoding stress enhances reality monitoring [27]. Here, CPS was applied after participants had performed or imagined performing brief actions. Although stress did not affect recognition of actions, it enhanced source recognition, that is, memory for whether the action had been imagined or performed. This form of source memory, however, relies more on the anterior frontal cortex than the hippocampus [28].

Post-encoding stress can also cause a dominant reliance on habit/stimulus-response (SR) memory over cognitive/context memory. Importantly, habit/SR memory relies on the dorsal striatum, whereas cognitive/context memory requires hippocampal engagement. Studies in rats show that stress applied pre-training or pre-retrieval biases towards the use of SR memory in dual-solution tasks; for a review, see [29]. Looking specifically at how post-encoding stress might affect human memory in such tasks, Goldfarb, Mendelevitch, and Phelps [30] asked participants to search for targets among distractors in the presence of two embedded memory cues, probabilistic SR associations and repeated contexts. Post-encoding stress in combination with heightened adrenergic activity during learning impaired consolidation and expression of context memory, while leaving the expression of SR memory unaffected.

Taken together, it appears that for neutral information, post-encoding stress enhances gist memory, but compromises associative binding and retention of detailed information. Although many studies have differentiated between neutral and emotional memories, fewer studies have looked at the specific components of a memory that might be affected by stress. The present study asked how stress affects the consolidation of contextual elements of episodic memories. Spatial context is a defining element of episodic memories and remembering the contextual elements of an experience or a memory’s source is crucial for applied settings, such as eyewitness testimonies. We presented participants with object images that were superimposed on scene images and asked about the impact of post-encoding stress on scene recognition. Specifically, we implemented a cold pressor stress (CPS) procedure that causes a painful stimulation that leads to an increase in cortisol and has been used successfully in prior studies in our lab [31,32]. At test, scene recognition could not be based on gist alone, but required the recollection of image details from the study phase because target scenes were presented alongside similar foil scenes from the same scene category. Since the hippocampus is crucial for binding items to contexts and for retaining fine-grained perceptual detail [33,34,35] but also highly sensitive to alterations in cortisol levels, we predicted that post-encoding stress would impair scene memory. We included male and female participants in our study, and predicted similar effects for both genders, because a meta-analysis found that participant sex did not moderate effects of post-encoding stress on episodic memory [5]. We included women regardless of hormonal contraceptive use and menstrual phase. However, because the use of hormonal contraceptives diminishes stress effects on memory [5], we asked women whether they used this birth control method.

## 2. Materials and Methods

### 2.1. Design and Participants

The study followed a 2 (post-encoding condition: cold pressor stress vs. warm water control) × 2 (gender) between-subjects factorial design. Fifty-six undergraduate students were recruited from Lehigh University and received course credit for participation (28 male, and 28 female students; mean age = 19.54, *SD* = 1.30, range: 18–22). We targeted a sample size of 50 participants based on previous studies assessing the effects of stress on memory consolidation [15,16], but purposefully oversampled in anticipation of dropouts. The experimental protocol was approved by the Lehigh University Institutional Review Board (IRB code: 136185-25), and informed consent was obtained from all participants prior to the study.

### 2.2. Materials

**Figure 1:**
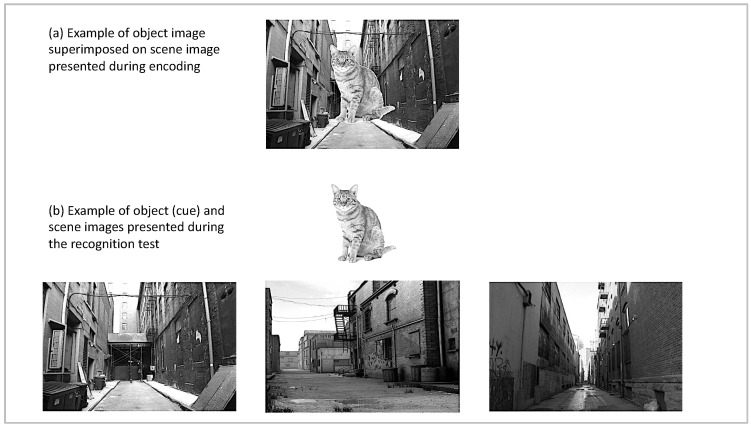
Examples for stimuli used during encoding (**a**) and test (**b**). Stimuli were presented in color in the study.

The stimuli consisted of 50 object images and 150 scene images selected from open access internet image databases. The scene images were comprised of three exemplars each for 50 scene categories (e.g., images of 3 different alleys). The object images depicted objects that can appear in a wide variety of contexts (e.g., a book can appear in an office, on a park bench, or in a store). Each object was superimposed onto the center of a scene image without substantially occluding it; see Figure 1 for an example and [36] for similar approach including faces as central elements. Object-scene category pairings were held constant across participants (e.g., the image of a cat always appeared on an image of an alley), but three encoding lists were created that varied in the specific scene image (e.g., specific alley) that was presented during encoding. Participants were randomly assigned to one of the lists. For the three-alternative forced-choice recognition test, previously encoded object images were presented on a white background centered at the top of the screen. The three images belonging to the scene category with which the object had been paired during encoding appeared below the object image. The exact placement of the target scene was counterbalanced across trials of the experiment.

### 2.3. Salivary Sampling and Biochemical Analysis

Saliva samples were collected using oral cotton swabs, which participants placed under their tongue for two minutes. After collection, swabs were placed into storage tubes and immediately transferred to a freezer where they were stored at −20 °C until analysis. Samples were sent to an independent commercial testing site (Salimetrics^®^, State College, PA, USA). Each sample was analyzed twice, and the mean value was used as the cortisol level per subject and timepoint in all analyses.

### 2.4. Procedure

**Figure 2:**
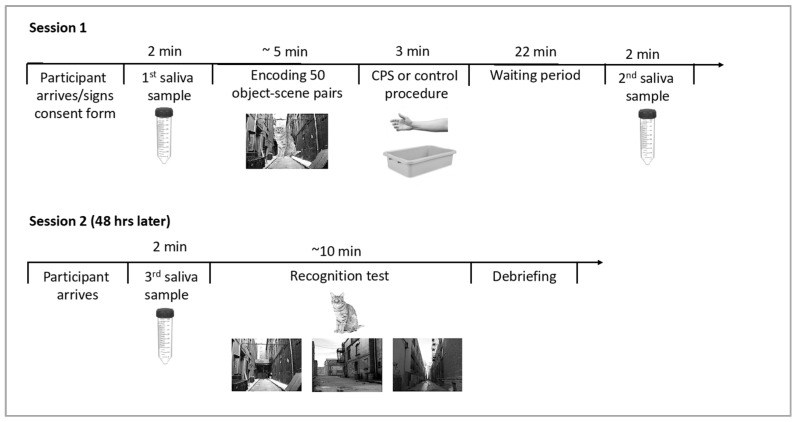
Schematic depiction of the experimental timeline with approximate durations of the different components.

A schematic outline of the experimental procedure can be found in Figure 2. Experimental sessions took place between 8 am and 11 am to take advantage of naturally high cortisol levels after awakening and their slow decline over the morning hours in order to induce cortisol levels that could compromise hippocampal processing. Participants were asked to refrain from eating, drinking, and exercise for one hour prior to the experimental sessions. Upon arrival for the first session, participants were asked to provide the first saliva sample as a baseline measure of cortisol levels. During the encoding phase, participants viewed 50 object-scene images one at a time in a random order. First, a fixation cross was displayed for 500 ms, followed by an object-scene pair. After 3 s, the image disappeared, and participants were asked to indicate whether the scene image was taken indoors or outdoors. This encoding task was implemented to ensure encoding of the background scenes. Immediately following the encoding phase, participants were asked to submerge their non-dominant arm up to their elbow in water for 3 min. In the CPS condition, the water bath contained ice-cold water (0–3 °C), and in the warm water control condition, lukewarm water was used (35–37 °C). Following the stress vs. control manipulation, participants were asked to rate stress and pain levels they had experienced during the water bath. Two independent scales were used to assess pain and stress levels, and scales ranged from 0 (“not stressful/painful at all”) to 10 (“extremely stressful/painful”). Additionally, females were asked about the use of hormonal birth control. Twenty-five minutes after the onset of the stress vs. control procedure, participants provided a second saliva sample. A 25-min delay was chosen because cortisol levels peak 20–30 min following the onset of CPS; e.g., [37]. Afterwards, participants were released, but returned to the lab 48 h later for the memory test session. Participants provided a third and final saliva sample prior to the beginning of the recognition test to control for stress effects on retrieval. For the recognition test, objects were presented alongside three highly similar scene images (one target scene, two foils). Stimuli were presented onscreen for a minimum of 2 s and remained visible until the subject made a recognition decision. Participants were asked to select the exact scene on which the central object had been presented during encoding by pressing a key on the keyboard corresponding to the left, center, or right image.

## 3. Results

### 3.1. Measures

The physiological measure of interest was the change in cortisol between the first sample taken upon arrival and the second sample taken after the exposure to the stress vs. control procedure. Further, we analyzed subjective ratings of pain and stress levels obtained from the questionnaire that was given immediately following the stress manipulation. For the recognition test, the critical measure was hit rates to target scenes.

### 3.2. Outliers

The data of four participants were excluded from analysis, because their memory scores were at or below chance performance (33%). Data of two other participants were excluded because of failure to detect cortisol levels in the baseline sample (one participant) or abnormally high baseline cortisol levels (one participant, more than 7 standard deviations above the mean). Thus, the final data set included data of 50 participants (12 male control, 13 male CPS, 13 female control, 12 female CPS, mean age = 20.00, *SD* = 1.51).

### 3.3. Analyses

#### 3.3.1. Cortisol Responses

**Figure 3:**
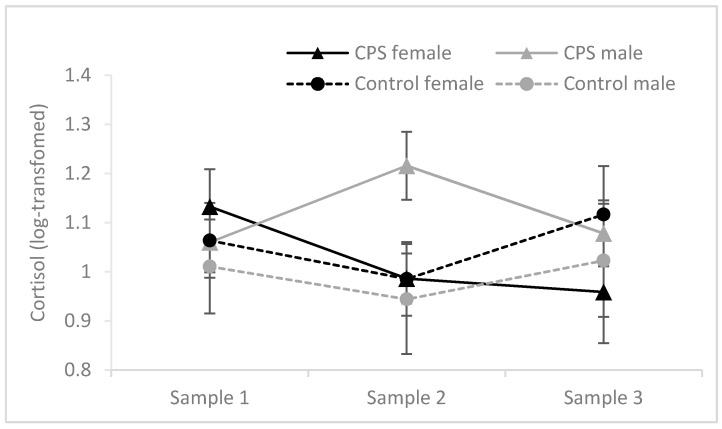
Salivary cortisol before encoding (Sample 1), after the stress/control manipulation (Sample 2) and before the final test in Session 2 (Sample 3) in males and females. Log-transformed cortisol values (original units: nmol/L) are shown. Error bars represent standard errors of means.

Cortisol values were not normally distributed and therefore log-transformed before analyses. Cortisol levels at baseline, post-stress and before retrieval in relation to condition and gender are depicted in Figure 3. The difference between Sample 1 and Sample 2 was analyzed with a 2 (gender) × 2 (condition: CPS vs. control) analysis of variance (ANOVA). This analysis revealed a significant main effect of gender, *F*(1, 46) = 6.754, MSE = 0.045, *p* = 0.013, η_p_^2^ = 0.128, and a significant interaction between gender and condition, *F*(1, 46) = 5.785, MSE = 0.045, *p* = 0.020, η_p_^2^ = 0.112. Critically, a significant difference between the stress and control group was found for male participants, *t*(18.420) = 2.363, *p* = 0.029, but not female participants, *t*(23) < 1. Cortisol levels measured upon arrival in Session 1 (Sample 1) and Session 2 (Sample 3) did not differ between the experimental groups (*F* ≤ 1.205, *p* ≥ 0.278).

#### 3.3.2. Memory Performance

**Figure 4:**
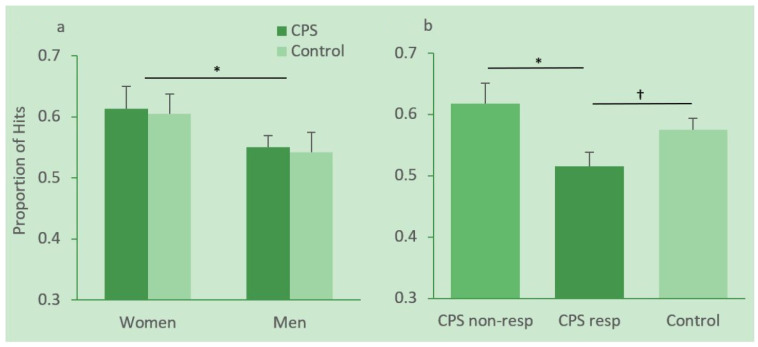
Proportion of hits for men and women in the cold pressor stress (CPS) and control group (**a**) and in CPS responders, non-responders and the control group (**b**). Error bars represent standard errors of means. * significant at the 0.05 level, ^†^ marginally significant.

Hit rates in women and men in the CPS and control condition are depicted in Figure 4a. Hit rates were analyzed with a 2 (gender) × 2 (condition) ANOVA. This analysis revealed a significant effect of gender, *F*(1, 46) = 4.19, MSE = 0.012, *p* = 0.05, η_p_^2^ = 0.083, with female participants performing significantly better than male participants. Neither the condition effect nor the gender × condition interaction reached significance, *Fs* < 1.

There are large interindividual differences in how people respond to stress, and not all participants in the CPS condition showed an increase in cortisol. We therefore divided participants in the CPS group into responders and non-responders. Following [38] responders were defined as participants who showed at least a 15.5% increase in cortisol after CPS (Sample 2) relative to their individual baseline (Sample 1). Hit rates in responders, non-responders and controls are depicted in Figure 4b. An independent-sample *t*-test revealed a significant difference in memory for context scenes between responders (*N* = 9, *M* = 0.52, *SD* = 0.07) and non-responders (*N* = 16, *M* = 0.62, *SD* = 0.13) in the CPS group, *t*(22.795) = 2.514, *p* = 0.019. The difference between responders and the control group (*N* = 25, *M* = 0.57, *SD* = 0.09) was marginally significant, *t*(32) = 1.70, *p* = 0.099. There were no female participants among the responders.

**Figure 5:**
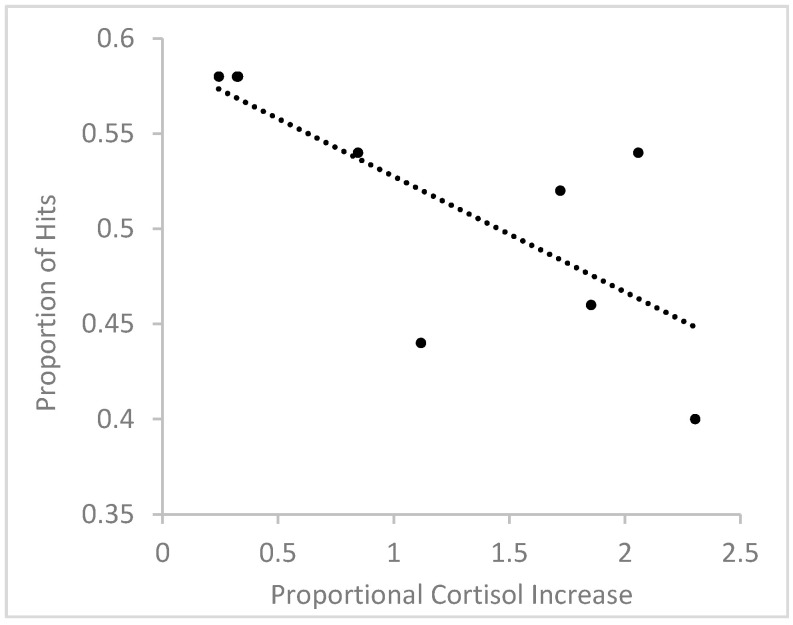
Relationship between relative cortisol increase and proportional hit rate in male responders.

In order to further assess the relationship between stress-induced cortisol increases and scene recognition, we correlated the relative increase in cortisol with the number of hits to target scenes in responders. To measure relative increase in cortisol, for each participant, we divided the cortisol difference between Sample 2 and Sample 1 by cortisol level in Sample 1. Using a relative change measure accounts for individual variations in baseline cortisol levels and stress responses [38,39]. A Shapiro-Wilk test showed that the distribution of relative cortisol increases did not significantly deviate from a normal distribution in the group of responders, *W*(9) = 0.893, *p* = 0.213, and therefore, data were not transformed. The relationship between relative cortisol increase and hit rates is depicted in Figure 5. The analysis revealed a significant negative correlation, *r*(9) = −0.733, *p* = 0.025, showing that the higher the relative cortisol increase, the fewer target scenes were correctly recognized. Using difference scores between log-transformed Sample 2 and Sample 1 cortisol levels instead of relative cortisol increases also resulted in a significant negative correlation between cortisol change and hit rates, *r*(9) = −0.746, *p* = 0.021.

#### 3.3.3. Subjective Ratings of Pain and Stress and Hormonal Contraceptive use

CPS induced significant increases in pain ratings (CPS: *M* = 6.78, *SD* = 2.01, control: *M* = 1.16, *SD* = 0.37), *F*(1, 46) = 191.957, MSE = 2.069, *p* < 0.001, η_p_^2^ = 0.807, and stress ratings, (CPS: *M* = 4.88, *SD* = 2.54, control: *M* = 1.44 *SD* = 0.58), *F*(1, 46) = 47.857, MSE = 3.154, *p* < 0.001, η_p_^2^ = 0.510, irrespective of gender (interactions, *F* ≤ 2.132, *p* ≥ 0.151). For stress ratings, gender was marginally significant, showing that females (*M* = 3.56, *SD* = 2.73) generally reported higher stress levels than males (*M* = 2.76, *SD* = 2.28), *F*(1, 46) = 191.957, *p* < 0.001, η_p_^2^ = 0.807. Pain and stress levels were not significantly correlated with the number of correctly recognized scenes in the CPS group, |*r*| ≤ 0.177 *p* ≥ 0.397. Three women in the CPS condition, and six women in the control condition used hormonal birth control methods.

## 4. Discussion

The present study asked how stress affects visual context memory. We applied stress after encoding to specifically target consolidation processes, that is, the conversion of short- into long-term memories, and selected a memory task that relies on hippocampal processing. We presented object images on scene backgrounds during encoding, and asked participants to recognize the target scenes among similar scenes in a delayed recognition test. Correct scene recognition requires retrieval of perceptual details and experiment-specific contextual components that are part of the relational representation the hippocampus establishes during encoding [33,34,35]. Previous studies have shown that post-encoding stress negatively impacts memory components that critically rely on the hippocampus, such as recollective experiences [16], reliance on cognitive/context memory [30], and differentiation between true and false memories [21]. Based on these findings, we had predicted that post-encoding stress would impair visual context memory. Contrary to these predictions, we did not find that stress exposure by itself affected recognition performance, that is, on a group level, CPS after encoding did not impair scene recognition in comparison to a control group. Despite the lack of a group difference, however, we observed impaired recognition of target scenes in participants who responded with an increase in cortisol to the CPS procedure, in comparison to non-responders. Other studies also report differential effects for responders and non-responders, e.g., [40,41,42], highlighting the importance of such a differentiation for assessing stress effects on memory. Importantly, the relative increase in cortisol was negatively correlated with hit rates in responders. This suggests that the long-term retention of visual context is related to physiological stress responsivity, but not the experience of stress alone. The lack of a relationship between subjective stress and pain ratings with memory performance further corroborates this conclusion. However, because of the small number of responders in our sample, these findings need to be treated as preliminary and interpreted with caution.

Although CPS was associated with elevated pain and stress levels in female participants, no significant increase in cortisol was detected in this group, replicating a previous study from our lab using a similar stress protocol [31]. In fact, none of the female participants in the current study was classified as a responder. The null effect likely reflects alterations of the hypothalamus-pituitary-adrenal axis activity by ovarian hormones. Hormonal contraceptives significantly reduce the cortisol response to a stressor [43,44]. However, this does not fully explain the lack of a cortisol response in our study because only 3 of the 12 women in the CPS condition used hormonal birth control methods. Endogenous fluctuations of hormone levels across the menstrual cycle also influence stress reactivity, with blunted cortisol responses in the follicular phase and male-comparable cortisol responses in the luteal phase [43,45]. Since we did not record menstrual phases, we cannot determine whether cortisol did not rise because the majority of the naturally cycling women in our study were in the follicular phase. Importantly, the effects of stress on memory are not exclusively determined by cortisol responsivity in women; e.g., [46]. Indeed, a meta-analysis on the effects of post-encoding stress on episodic memory identified neither participant sex nor cortisol response as moderating factors [5]. However, no cortisol-independent stress effects were found in the current study, and none of the responders were women. Many studies assessing the effects of stress on cognition exclude women but there is clearly a need for future research to unravel the complex relationship between sex and stress hormones and cognition in women.

After our data collection was complete, we became aware of a recent study that used a somewhat similar design but obtained opposite results [39]. In this study, participants encoded images of everyday objects. Two days later, the same images, lure images of perceptually similar objects or images of entirely new objects were presented in a recognition test, and participants had to indicate for each image whether it was old, similar or new. Either immediately after encoding or right before the recognition test, the Trier Social Stress Test or a control procedure was implemented. In contrast to previous studies, stress experienced right before retrieval did not affect memory. Stress during consolidation increased “similar” responses to lure images but did not affect “old” responses to repeated targets. Based on this observation, the authors suggest that stress improved the retention of perceptual detail, which allowed participants to determine that similar images were different from target images in the recognition test (for a similar result but restricted to negative images, see [47]). This interpretation, however, is complicated by the fact that the consolidation-stress group also called entirely new items similar more often than the control group. Since discrimination indices that account for both correct responses to similar lures and false alarms to new foils are missing, it is not entirely clear whether the consolidation-stress group indeed retained more perceptual detail or simply used the “similar” response category more liberally across all lure types. This is important because stress should specifically impact detail but not gist memory, as outlined in the Introduction. The discrepancy in results between Jiang et al.’s and our study could also be due to the difference in stress hormone levels. We tested participants in the morning hours when baseline cortisol is naturally high whereas Jiang et al.’s study was carried out in the afternoon. Additionally, our average cortisol increase in responders was almost three times as large as the one observed by Jiang et al. (0.42 compared to 0.15 mg/dL). Thus, in line with the inverted U-shape relationship between cortisol levels and hippocampal processing, the lower levels might have benefitted, whereas the higher levels might have impaired memory performance. Furthermore, stress might affect central and peripheral elements of a display differently. Jiang et al. tested memory for objects (presented on white backgrounds), whereas we specifically tested memory for background scenes. Under normal conditions, central elements are better retained over time, whereas peripheral elements are lost more rapidly [48]. Stress might potentiate this difference by selectively strengthening the consolidation of central elements. This could explain why Jiang et al. found enhancing and we found impairing effects of stress on visual recognition memory.

One limitation of our study is that it did not simultaneously assess memory components that could be differentially affected by stress. For instance, we did not test memory for central objects and memory for specific object-scene associations nor did we differentiate between familiarity and recollection-based processes. Based on [39] and the current study, we might expect that stress enhances the consolidation of central at the cost of peripheral details. In terms of object-scene associations, it would be interesting to differentiate between scene categories and specific scene exemplars, to assess which level of detail is affected by stress.

A recent framework highlights context as a crucial factor determining the directionality of post-encoding stress effects on episodic memory. According to the contextual-binding account, a stressful experience will create a particularly salient episodic memory, for the stressor and the physical and mental context in which it occurred. Material that is learned just prior to and in the same context as the stressful experience will benefit from the stress-enhanced saliency of the context [49]. In line with these predictions, a recent study showed that CPS improved recollection of negative and neutral images when CPS was applied in the same context in which the images had been encoded in comparison to a context change condition in which CPS was applied in a different building and as part of a seemingly different study [49]. In our study, the encoding and CPS took place in adjacent rooms in the same lab in the context of the same study. Perhaps this change of context was sufficient to obliterate potential stress-enhancing effects on scene recognition. However, the contextual-binding account cannot explain the cortisol-related effects on memory. Alternatively, these effects could be explained with a memory integration framework [50]. It could be the case that responders experienced a significant change in their internal state after encoding, and that this state information was then integrated into their object-scene memory. Since the post-encoding internal state was not reinstated before test, their memory suffered in comparison to non-responders and controls who did not experience a significant state change.

Independent of stress exposure, we found overall better context memory in women compared to men. This effect is in line with a recent meta-analysis showing an overall female advantage in episodic memory [51]. However, in the meta-analysis, sex differences were moderated by the to-be-remembered material, with women exhibiting better memory for verbal tasks, nameable images and locations, and men outperforming women in memory for abstract images and routes. In our study, women showed better memory for background scenes, which share features with nameable images and locations. Other studies have also found better context learning and retention in female participants [30]. The underlying nature of these sex differences is not well understood, but they likely reflect influences of endogenous sex hormones, environmental factors and their interaction [51].

Taken together, our study shows that stress negatively affects the consolidation of visual context memories in participants who responded with an increase in cortisol to a physiological stressor. This finding suggests that stressors that follow an otherwise neutral event can influence the subsequent retrieval of event details. This should be kept in mind when evaluating the credibility of eyewitness accounts. The retrieval of contextual details might be impaired by post-event stressors, even if those stressors are entirely unrelated to the event in question.

## 5. Conclusions

The present study shows that post-encoding cold pressor stress negatively affected the recognition of background scenes, but only in participants who reacted with an increase in cortisol to the stressor. Because of the small number of responders, this finding needs to be interpreted with caution but provides preliminary evidence that stress-induced cortisol increase negatively affects the consolidation of contextual elements of episodic memories.
